# A new species of the cicada genus *Cicadatra* Kolenati, 1857 (Hemiptera, Cicadidae) from Pakistan with a key to the known species of Pakistani *Cicadatra*

**DOI:** 10.3897/zookeys.174.2299

**Published:** 2012-03-09

**Authors:** Zubair Ahmed, Allen F. Sanborn, Muhammad Atique Akhter

**Affiliations:** 1 Department of Zoology, Federal Urdu University of Arts, Sciences & Technology, Karachi, Pakistan; 2 Department of Biology, Barry University, 11300 NE Second Avenue. Miami Shores, FL 33161-6695, USA; 3 Department of Zoology, University of Karachi, Karachi, Pakistan

**Keywords:** *Cicadatra* species, taxonomy, Cicadidae, morphology

## Abstract

A new species of cicada, *Cicadatra ziaratica* **sp. n.**, is described from Pakistan. Male genitalia, timbal and opercula are described and illustrated as important diagnostic characters. Biological notes are also provided. A key to the known *Cicadatra* of Pakistan is provided.

## Introduction

Species of the genus *Cicadatra* Kolenati, 1857 exhibit variability in several morphological characters. As a result, there is still much to learn about the distribution and composition of *Cicadatra* species across the range of the genus. Studies of *Cicadatra* species in the Middle East and Asia continue to illustrate the lack of knowledge about the species of this widespread genus. However, there have been some more recent studies which provide detailed analyses of the morphological characters of generally new species from this region (Dlabola 1960, 1970, 1979, 1981, Dlabola and Heller 1962, Linnavouri 1962, Boulard 1977, Melichar 1896, Mirzayans 1995, Mozaffarian and Sanborn 2010, Mozaffarian et al. 2010, Ahmed and Sanborn 2010, Ahmed et al. 2010) including the first checklists of the genus for Iran (Mozaffarian and Sanborn 2010) and Pakistan (Ahmed and Sanborn 2010).

The first checklist of the Pakistani cicada fauna was recently produced by Ahmed and Sanborn (2010). They determined that 29 known species inhabited Pakistan at the time with the first records of seven species and the descriptions of four new species included in the total (Ahmed and Sanborn 2010). They listed seven species of *Cicadatra* for Pakistan, four of which represented new records for the country. Later that same year, Ahmed et al. (2010) described a new species of *Cicadatra* from Pakistan along with sound analysis and DNA sequencing of the species. The current work represents another new species that was collected during recent fieldwork. A more complete knowledge of the Pakistani cicada fauna will only be obtained with continued field research and studies of existing collections.

The present species is described from Pakistan as new to science, and is known only from the Ziarat District, Balochistan Province. Notes on the biology of this new species are also provided along with a key to differentiate the species from the known Pakistani *Cicadatra*.

## Materials and methods

Specimens were captured during June 2010 and June 2011 in Balochistan Province, Pakistan. Terminology follows Moulds (2005). Measurements were made with Vernier calipers or a Wild Heerbrugg 12034 binocular microscope. Specimens are deposited in the collections of the Natural History Museum, University of Karachi, Pakistan (NHMK) and Zubair Ahmed Collection, Pakistan (ZACP).

## Results and discussion

### Cicadatra ziaratica

Ahmed, Sanborn & Akhter sp. n.

urn:lsid:zoobank.org:act:04C75272-8CDE-4677-A6EE-798512C83B53

http://species-id.net/wiki/Cicadatra_ziaratica

Figs 1 –8

#### Type locality.

Pakistan, Balochistan Province, Khotal Chehri, District Ziarat.

#### Type specimens.

Holotype male, pinned. Original label: “Pakistan, Balochistan Province, Khotal Chehri, District Ziarat, 7.vi.2010, Collector, Zubair Ahmed”, “HOLOTYPE / Cicadatra ziaratica / Ahmed, Sanborn & Akhter” [handwritten label] (NHMK); one male paratype, “Pakistan, Balochistan Province, Khotal Chehri, District Ziarat, 7.vi.2010, Collector, Zubair Ahmed”, “PARATYPE / Cicadatra ziaratica / Ahmed, Sanborn & Akhter” [handwritten label]; three male paratypes, “Pakistan, Balochistan Province, Khotal Chehri, District Ziarat, N, 3.vi.2011, Collector, Zubair Ahmed “PARATYPE / Cicadatra ziaratica / Ahmed, Sanborn & Akhter” [handwritten label] (ZACP).

#### Diagnosis.

The new species appears to be most allied morphologically to *Cicadatra lorestanica* Mozaffarian and Sanborn 2010 from Iran and *Cicadatra karachiensis* Ahmed et al. 2010 from Pakistan. The new species can be distinguished by the upper lobe of the pygofer being ill-defined in *Cicadatra ziaratica* whereas it is a finger-like extension in *Cicadatra lorestanica*. The aedeagus of *Cicadatra lorestanica* has a curved, bifold, sclerized, hook-like process and two lateral spiny appendages while the aedeagus of *Cicadatra ziaratica* has a long, subapical spine, a dorsal spine along sclerized teeth-like process, a lateral spine and a ventral semicircular toothed process. In *Cicadatra karachiensis* the upper lobe of the pygofer is rounded, the aedeagus has a long upturned flap with 11 aedeagal spines and the hind wing has five apical cells instead of the six found in *Cicadatra ziaratica*. The species are similar in possessing a mesonotum with two lines but the degree of curvature is slightly variable. Fore wings with radial and radiomedial crossveins at bases of the 2^nd^ and 3^rd^ apical cells infuscated in *Cicadatra ziaratica* but lacking infuscation in *Cicadatra lorestanica* and *Cicadatra karachiensis*. The timbal cover of *Cicadatra karachiensis* is reduced and ventral to the majority of the timbal while the timbal cover in *Cicadatra ziaratica* and *Cicadatra lorestanica* covers more than half the timbal and is centrally located over the timbal. Finally, the timbal has 9 ribs in *Cicadatra ziaratica* and *Cicadatra karachiensis* but 11 ribs in *Cicadatra lorestanica*. The remaining species known to inhabit Pakistan can be distinguished using the key. There are insufficient data to perform a molecular phylogenetic analysis of the Pakistani *Cicadatra* species as genes from a limited number of species have been sequenced (Ahmed et al. 2010).

#### Description.

***Male.*** General color of body black with olive to ochraceous markings and white pile.

Head black with white pile particularly on posterior edge, head including eyes as broad as mesonotum; eyes brown, varying from light to dark in different specimens; ocelli orangish, piceous in some specimens; postclypeus black with a central sulcus and obvious transverse grooves, dense pile lateral of grooves, gena and lorum black with dense white pile; rostrum light ochraceous at base, darker towards apex, strongly passing intermediate coxae; labrum with sparse white pile laterally and on apex; antennae dark brown, apical segment faint yellow, vertex black, supra-antennal plate reaching eyes, black, light band at medially in two paratypes.

Pronotum black, brown in some paratypes, with median black biconcave mark containing a light olive green median fascia, an olive green patch crossing ambient fissure posterolateral to each side of median fascia, black mark continues around disc in ambient fissure and across lateral pronotal collar to the lateral angle; paramedian and lateral fissures variably marked with dark brown to black, pronotal collar black anteriorly and olive green across posterior half of lateral angles and posterior margin, ochraceous in some paratypes, white dense pile present on ambient, paramedian and lateral fissure and scattered pile on disc, pile reduced in some paratypes; mesonotum black or dark brown, with ochraceous J-shaped mark along parapsidal suture, mark triangularly shaped at base in some paratypes; cruciform elevation olive green (brown in some paratypes) medially, darkening to black in anterior arms; metanotum olive green (brown in some paratypes); thoracic sternites black with dense white pile, ochraceous in different specimens; some specimens with dark marking on basisternum 2, epimeron 2, katepisternum 2, and episternum 3.

Fore coxae light olive to ochraceous with black linear marking, middle coxae light ochraceous with broad dark anterolateral surface, hind coxae light ochraceous with darker laterally; fore and middle trochanter olive to ochraceous with a dark brown area at middle with white pile; fore femorae dark brown with white pile and light areas on ventral apex with strongly angled primary spine, erect secondary spine and a small angled apical spine; middle femora dark brown with a yellow area at ventral and apex with dense white pile, hind femora dark brown with yellow area on base and apex; fore tibiae dark brown lighter at apex, middle tibiae dark brown with white pile and yellow at lateral, hind tibiae yellow, half dark brown with five brown tibial spurs and sparse white pile; tibial spurs and combs brown, darker towards their apices; tarsi black; pretarsal claws dark brown.

Fore wings hyaline with faint yellow and brown venation, radial (r) and radiomedial (r-m) crossveins at bases of apical cells 2 and 3 darkly infuscated, infuscation on r-m absent or reduced in some paratypes, basal call twice as long as wide; fore wings with 8 apical cells, basal membrane light reddish; hind wings with faint yellow venation, light grey infuscation around anal veins 2 and 3 (2A and 3A), hind wings with 6 apical cells.

Male opercula light brown with black spot on lateral base and rather dense white pile, rounded, and slightly overlapped, not meeting medially in paratypes, meracanthus triangular, light ochraceous with black spot at base.

Abdominal tergites black with white pile more or less located near the anterior edge of each tergum, tergites 2–7 with a light area on posterior except median part, timbal cavity exposed; timbal cover incomplete covering about half the timbal, black or dark brown with white pile, timbal with 9 ribs; abdominal sternites brown with dense white pile, epipleurites dark brown with dense white pile.

Male pygofer dark brown with scattered pile, dorsal beak pointed, upper lobe of pygofer rounded, basal lobe of pygofer appears as a bud like projection beneath the upper lobe; uncus very short; claspers tapering to a point, curved slightly laterad, close to each other at base; aedeagus with theca curved, a lateral scleritized, serrate appendages, a long, subapical spine, a ventral scleritized, rounded serrate process, a lateral and a median long spine.

***Female.*** Unknown.

**Figure 1.**
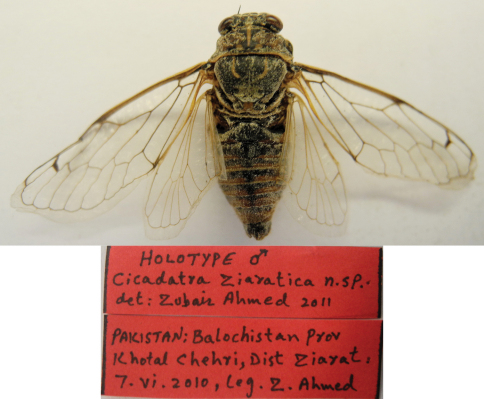
Holotype Male, *Cicadatra ziaratica* sp. n.

**Figures 2–8.**
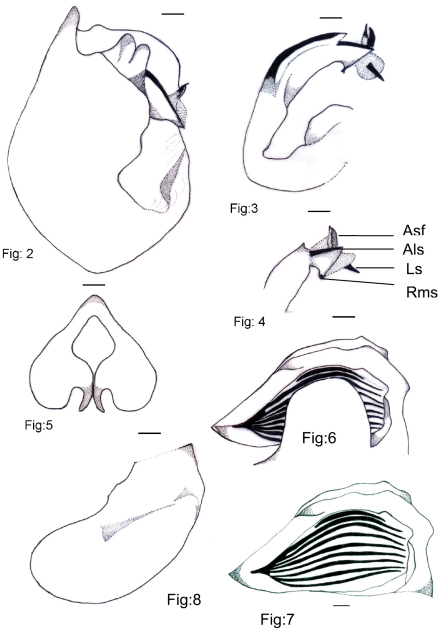
*Cicadatra ziaratica* sp. n., 2 Male pygofer lateral view 3–4 Aedeagus 5 Claspers 6 Timbal cover 7 Timbal 8 Operculum. Scale lines = 0.6 mm. **Asf** Aedeagus with serrated flap **Als** Aedeagus long spine **Ls** Lateral spine **Rss** Row of middle spines

#### Etymology.

 The species is named for the district of Balochistan from which the type series was collected.

#### Measurements

**(mm).** N=5 males, mean (range). Length of body: 16.9 (16.0–18.0); length of fore wing: 20.2 (19.0–22.0); width of fore wing: 6.7 (6.1–7.0); width of head including eyes: 4.6 (4.0–5.0); width of pronotum including paratota: 5.7 (5.5–6.0); width of mesonotum: 5.1 (4.8–5.5).

#### Biological notes.

All specimens were collected during 2010 and 2011 in the vicinity of Ziarat between 3 June–7 June. The cicadas emerged among wild grasses based on the location of the emergence holes. Adult males called from these same grasses as well as from shrubs including *Peganum harmala* L.

##### Key to the males of Cicadatra of Pakistan

**Table:** 

1	Body length >24 mm, prontoal collar almost or entirely black	2
–	Body length <24 mm, pronotal collar heavily marked with ochracous, olivaceous or tawny	4
2	Pronotal disk black	*Cicadatra acberi* (Distant, 1888)
–	Pronotal disk ochraceous	3
3	Supra-antennal plate, cruciform elevation, and costal margin tawny	*Cicadatra persica* Kirkaldy, 1909
–	Supra-antennal plate and cruciform elevation black, costal margin castaneous	*Cicadatra gingat* China, 1926
4	Head castaneous	*Cicadatra raja* (Distant, 1906)
–	Head black	5
5	Radial and radiomedial crossveins not infuscated	6
–	Radial and/or radiomedial crossveins infuscated	7
6	Pronotum castaneous marked with black, cruciform elevation black, male opercula overlapping medially, small marginal spot on hind wing	*Cicadatra sankara* (Distant, 1904)
–	Pronotum dark ochraceous marked with black, cruciform elevation marked with ochraceous, male opercula almost meeting medially, hind wing hyaline	*Cicadatra karachiensis* Ahmed, Sanborn and Hill, 2010
7	Postclypeus black	*Cicadatra ziaratica* sp. n.
–	Postclypeus tawny marked with castaneous or black	8
8	Postclypeus with transverse grooves black	*Cicadatra walkeri* Metcalf, 1963
–	Postclypeus with medial castaneous or black stripe	*Cicadatra xanthes* (Walker, 1850)

## Supplementary Material

XML Treatment for Cicadatra ziaratica
